# Supplementary material for: Optimization of cerebrospinal fluid microbial DNA metagenomic sequencing diagnostics
Source: Sci Rep. 2022 Mar 1;12:3378. doi: 10.1038/s41598-022-07260-x (PMC8888594; doi:10.1038/s41598-022-07260-x)
Supplement: Supplementary file 1 — Supplementary Information 1. [file 41598_2022_7260_MOESM1_ESM.pdf]

Additional figure 1. Enterovirus samples

Sample 8 – 1,455,168 total classified reads

Viruses Hits	- Taxid	% (RNA)	Reads (RNA)
Enterobacteria phage T4 sensu lato	348604	4.2333	61783
Enterovirus B	138949	0.1643	2398
Escherichia virus M13	1977402	0.1433	2092
Enterobacteria phage RB3	31533	0.0843	1231
Escherichia virus T7	1985738	0.0276	403
Pseudomonas phage PPpW-4	1279083	0.0246	359
Shigella phage SHFML-26	1863009	0.0166	242
Escherichia virus P1	10678	0.0094	137
Enterobacteria phage 933W sensu lato	374810	0.0035	51
Escherichia phage vB_EcoM-UFV13	1815590	0.0034	49
Enterobacteria phage RB32	45406	0.0026	38
untyped human enterovirus	235406	0.0023	33
Escherichia virus Ime09	1054834	0.0010	15
Escherichia virus Lambda	10710	0.0009	13
Escherichia phage UFV-AREG1	1837867	0.0009	13
Enterobacteria phage RB27	69609	0.0008	11
Escherichia phage slur07	1720500	0.0008	11
Escherichia virus AR1	1914199	0.0008	12
Shigella virus Pss1	1551641	0.0007	10
Escherichia phage HK639	906669	0.0005	8
Enterobacteria phage RB51	10693	0.0005	7
Escherichia virus E112	1495285	0.0005	8
Salmonella phage RE-2010	929814	0.0004	6
Stx converting phage vB_EcoS_ST2-8624	1792289	0.0004	6

Sample 9 – 1,875,002 total classified reads

Viruses Hits	- Taxid	% (RNA)	Reads (RNA)
Escherichia virus T7	10760	0.0584	1101
Enterovirus B	138949	0.0316	595
Enterobacteria phage T4 sensu lato	348604	0.0135	254
Enterovirus A	138948	0.0119	224
BeAn 58058 virus	67082	0.0052	98
Escherichia virus M13	1977402	0.0005	9

Results of viral species detected in RNA sequencing datasets of sample 8 and sample 9 in PaRCA. Reads mapping to enterovirus was found amongst virus lists in both specimens.

Additional figure 2. Detected pathogens in bioinformatic classifiers

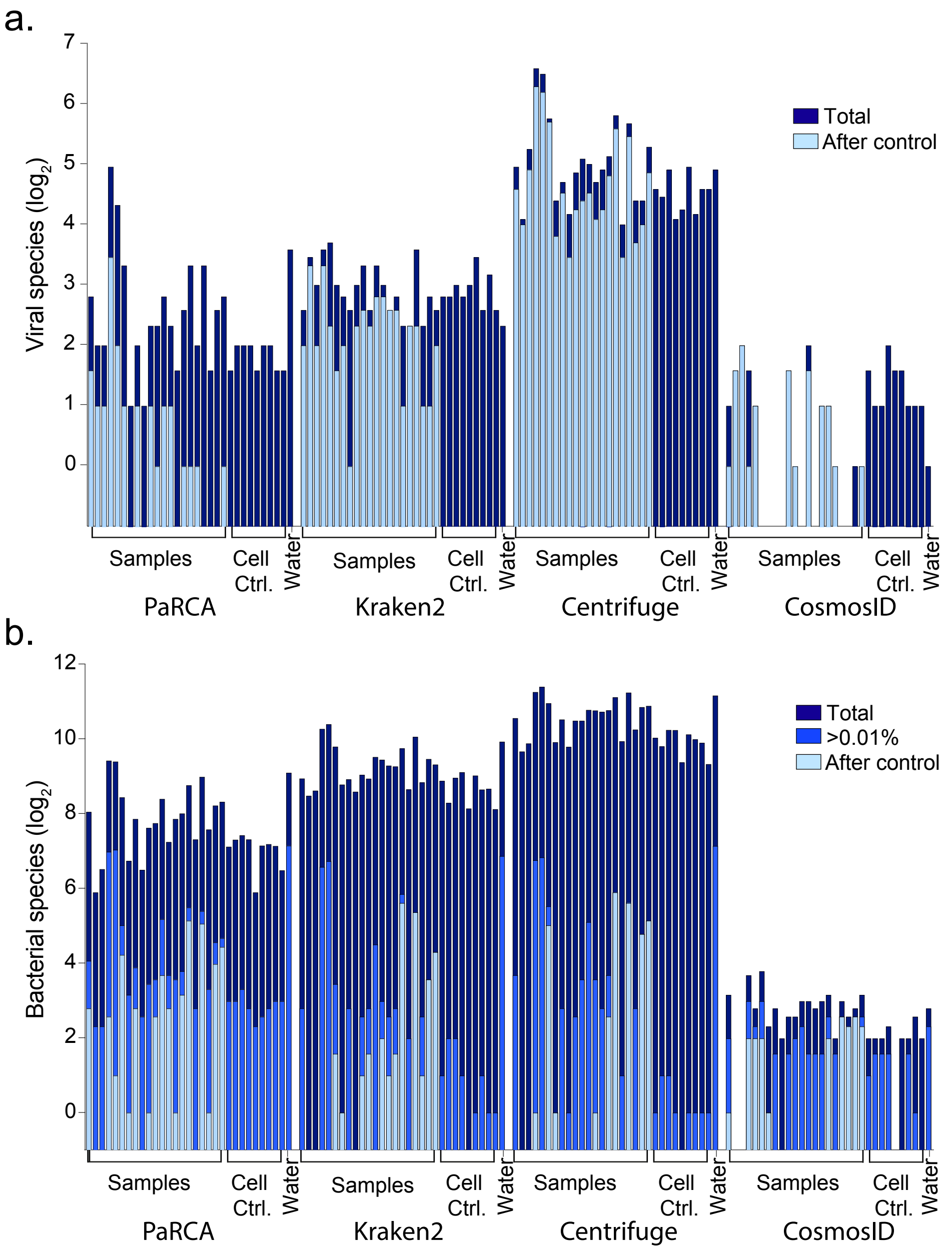

Number of viral (a) and bacterial species (b) classified in each of the samples and controls using the different bioinformatic classifiers. Dark blue bars show the total number of species classified, bright blue bars show the number of bacterial species over the fraction cutoff (0.01% of the dataset), light blue bars show number of species not removed using controls.

Additional figure 3. Coverage density plot of microbial species in CSF samples

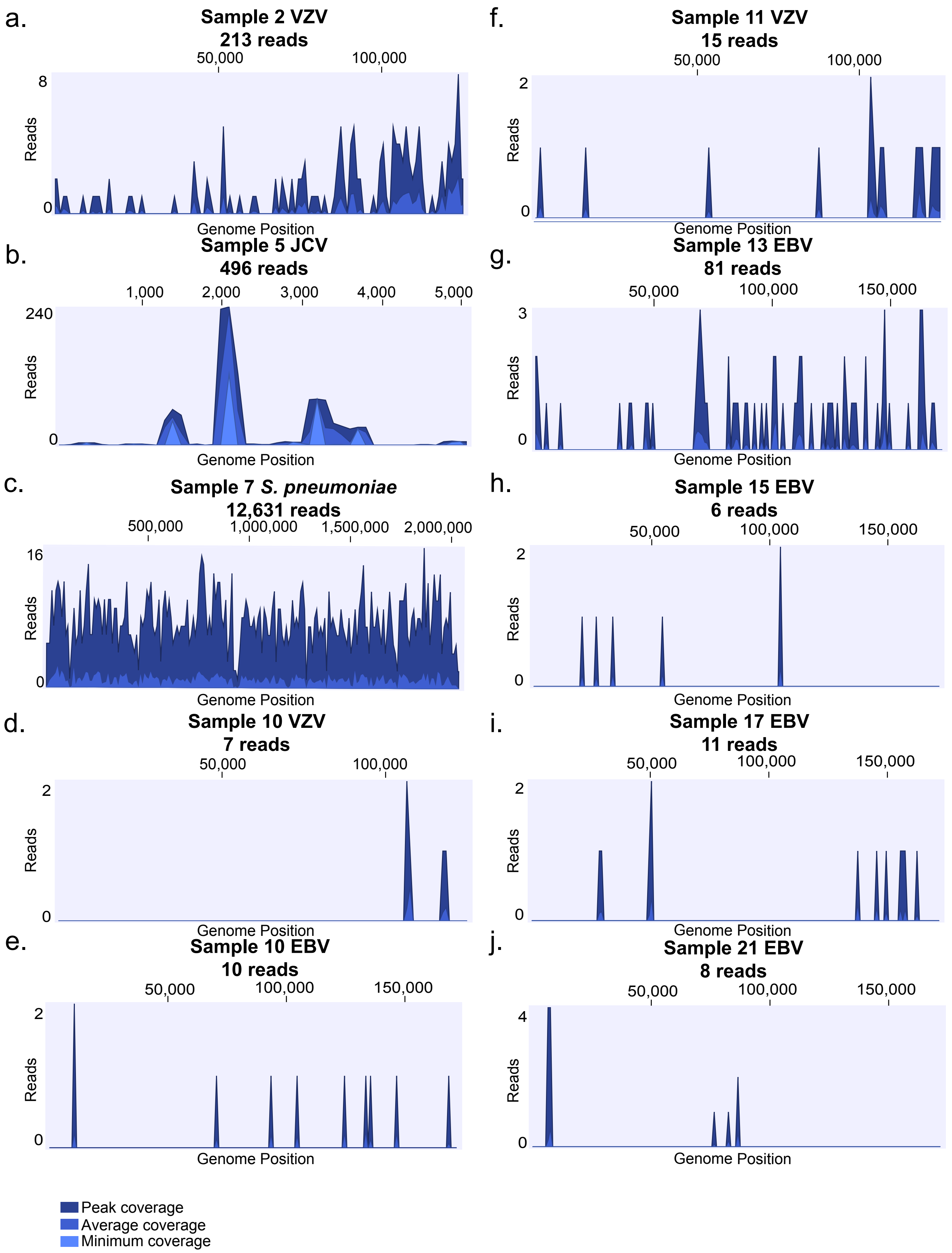

Reads from samples not shown in main figure mapped to reference genomes of (a) VZV (NC_001348), (b) JCV (NC_00196), (c) S. pneumoniae (NC_003098), (d, f) VZV (NC_001348), and (e, g-j) EBV (NC_007605) using CLC Genomics Workbench. Number of reads (y-axis) at each nucleotide position of the genome (x-axis) depicted in blue. Dark blue represents peak, bright blue average and light blue minimum coverage for respective sections of the genome.

Additional figure 4. Cell control coverage density plot and reproducibility

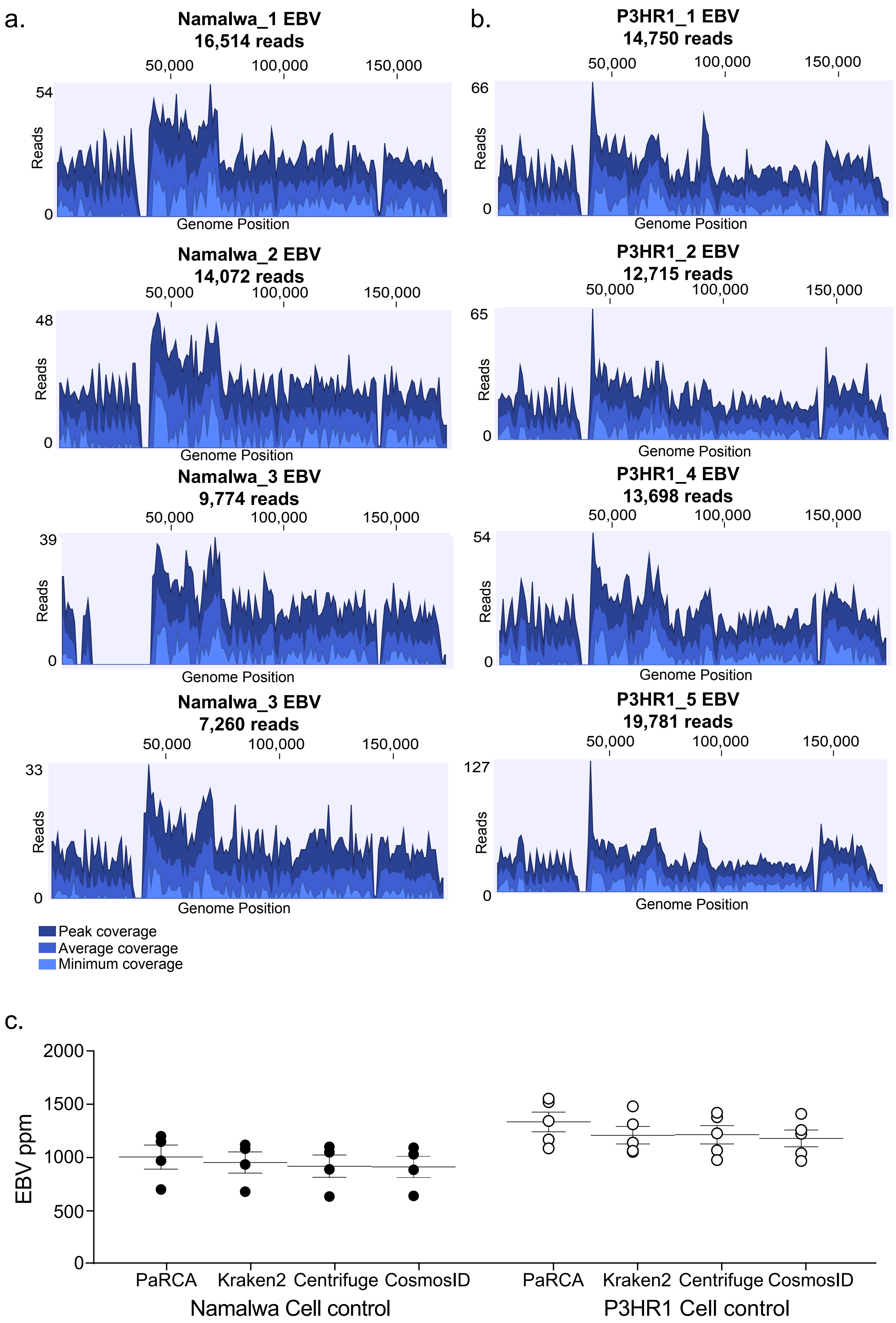

Coverage analysis of EBV reads detected in cell controls Namalwa (a) and P3HR1 (b) mapped to EBV reference genome (NC_007605) using CLC Genomics Workbench. Number of reads (y-axis) at each nucleotide position of the genome (x-axis) depicted in blue. Dark blue represents peak, bright blue average and light blue minimum coverage for respective sections of the genome. EBV read fraction of sequencing library shown as parts per million reads (ppm) in each of the cell line controls for each of the bioinformatic classifiers (c), n=4 (Namalwa) or n=5 (P3HR1). Kruskal-Wallis test with Dunn's multiple comparisons show no significant difference between the pipelines.

Additional figure 5. Coverage analysis for unexpected findings

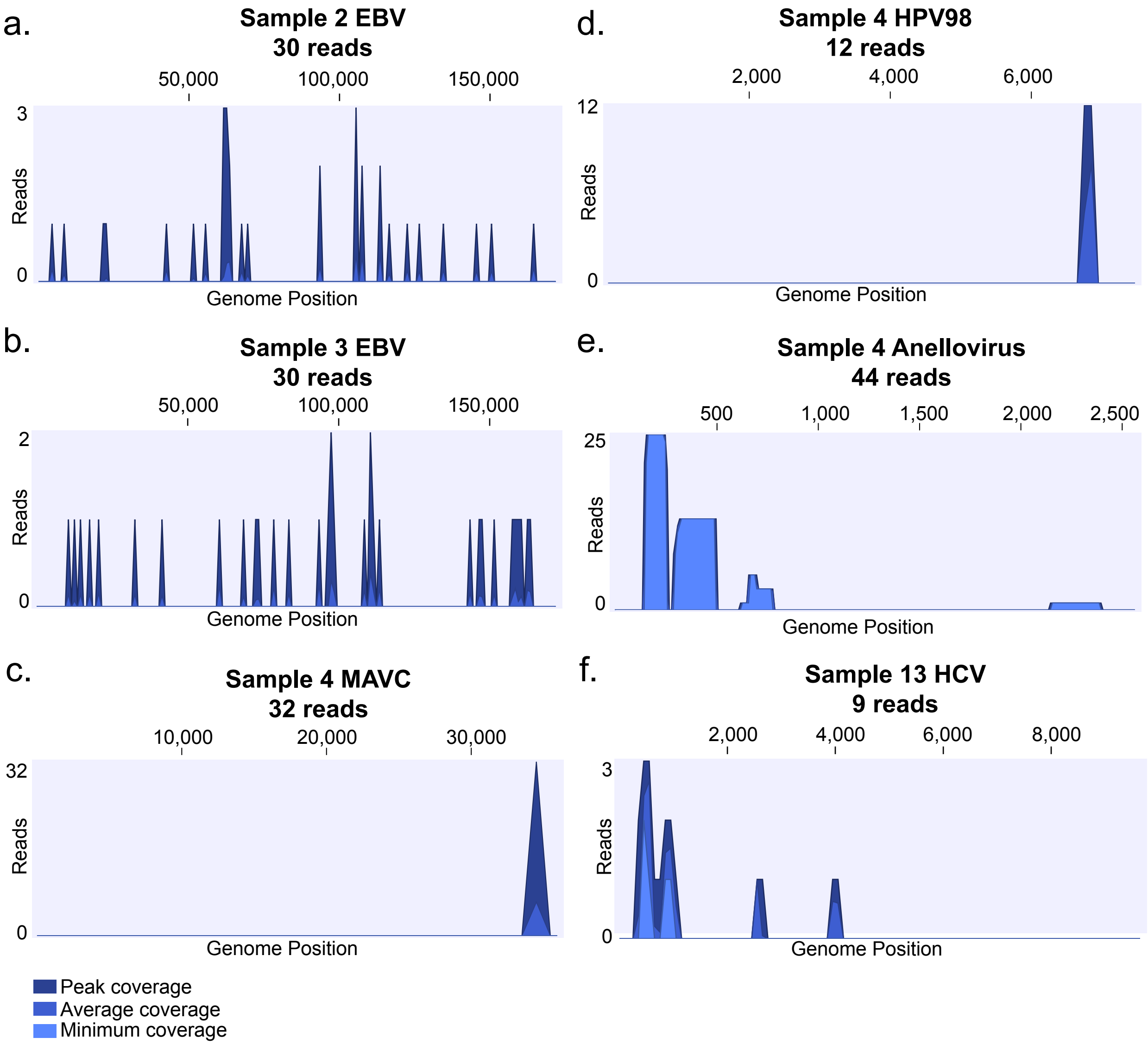

Reads from samples with ambiguous findings mapped to reference genomes of EBV NC_007605 (a-b), Human Mastadenovirus C (MAVC) NC_001405 (c), Human Papillomavirus 98 (HPV98) FM_955837.2 (d), Anellovirus MH_649255.1 (e), and HCV NC_004102.1 (f), using CLC Genomics Workbench.
