# Supplementary material for: Optimization of cerebrospinal fluid microbial DNA metagenomic sequencing diagnostics
Source: Sci Rep. 2022 Mar 1;12:3378. doi: 10.1038/s41598-022-07260-x (PMC8888594; doi:10.1038/s41598-022-07260-x)
Supplement: Supplementary file 2 — Supplementary Information 2. [file 41598_2022_7260_MOESM2_ESM.docx]

**Additional methods**

**Quantitative PCR**

DNA was extracted from 200 µl of cerebrospinal fluid using the MagNA Pure DNA Isolation Kit I (Roche Diagnostics) on the MagNA Pure LC automated extractor. Enterovirus RNA was extracted using MagNA Pure Total Nucleic Acid Isolation kit. Quantitative PCR was performed using TaqMan Universal PCR mastermix on QuantStudio DX (Applied Biosystems). Quantification standards consisted of plasmids (pUC57) with inserts of viral sequence. Primers and probes used are described in Table 1.

**Filmarray**

The FilmArray Meningitis-Encephalitis Panel was used according to the manufacturer’s instructions (bioMérieux).

**Cerebrospinal fluid culture**

BacT/ALERT culture system (bioMérieux) was used for CSF cultures. Positive samples were subcultured on solid medium and cultured bacteria was typed by matrix-assisted laser desorption/ionization time-of-flight on a VITEK MS RUO instrument (bioMérieux).

**16S rRNA gene Sanger Sequencing**

DNA was extracted from 400 µl of cerebrospinal fluid using the MagNA Pure Compact DNA Isolation Kit I (Roche Diagnostics) on the MagNA Pure Compact automated extractor. PCR targeting the 16S rRNA gene (Table 1) was carried out as earlier described [1]. Briefly, PCR mixtures were illuminated with UV light at 312 nm (8 W) for 15 min in the reaction tubes to remove DNA contaminations in the mixtures. Extracted DNA was added and amplified using DNA Thermal Cycler 2400 (Applied Biosystems), with 40 cycles of 15 s at 94°C, 30 s at 55°C and 1 min at 72°C, followed by 10 min at 72°C. Aliquots of PCR products were separated in agarose 2% w / v gels stained with ethidium bromide. DNA bands of the expected size (766 bp) were cut from the gel, purified with a QiaQuick Gel Extraction kit (Qiagen), sequenced using the ABI PRISM Big Dye Terminator Cycle Sequencing Ready Reaction kit, (Applied Biosystems). Sequencing products were analyzed using an ABI PRISM 310 Genetic Analyser (Applied Biosystems). After DNA sequence editing, sequences were aligned to BLAST.

**Table 1.** Primers and probes.

| Virus | Primer/Probe | Sequence (5’-3’) | Position | Reference |
| --- | --- | --- | --- | --- |
| Epstein-Barr Virus (quantitative PCR) | Forward  Reverse  Probe | GGAACCTGGTCATCCTTTGC  ACGTGCATGGACCGGTTAAT  FAM-CGCAGGCACTCGTACTGCTCGCT-BHQ1 | 4679-4698  4733-4752  4700-4722 | [2] |
| JC polyomavirus (quantitative PCR) | Forward VP2  Reverse VP2  Probe VP2 | CTGAACCAAAAGCTACATAGGTAAGTAATGT  CTAGGTCCCCCAAAAGTGCAA  FAM-AGGTTCATGGGTGCCGC-MGB | 473-503  559-539  520-539 | [3] |
| Human Herpesvirus 1 (quantitative PCR) | Forward  Reverse  Probe | GCAGTTTACGTACAACCACATACAGC  AGCTTGCGGGCCTCGTT  FAM-CGGCCCAACATATCGTTGACATGGC-MGB | 54253-54278  54162-54178  54228-54252 | [4] |
| Human Herpesvirus 3 (quantitative PCR) | Forward  Reverse  Probe | TGCAGGGCATGGCTCAGT  CCCAAGAACCACATGTCCAAC  FAM-CGCGGTCCCAAGTCCCTGGA-BHQ1 | 59049-59066  59099-59119  59070-59089 | [5] |
| *Streptococcus pneumoniae* (16S rRNA gene Sanger sequencing) | Forward  Reverse | CGGCGTGCCTAATACATGCAAGTCG  GGACTACCAGGGTATCTAAT | 15190-15212, 1697117-1697139, 1794667-1794689, 1856592-1856614  1696369-1696388, 1793919-1793938, 1855844-1855863, 15941-15960 | [1, 6, 7] |
| Enterovirus (quantitative PCR) | Forward 1  Forward 2  Forward 3  Forward 4  Reverse  Probe | AGGTGTGAAGAGCCTATTGAGCTA  TGGTGTGAAGAGTCTATTGAGCTA  TGGTGCGAAGAGTCTATTGAGCTA  TGGTGCGAAGAGCCTATTGAGCTA  GGACACCCAAAGTAGTCGGTTC  VIC-CGGCCCCTGAATGCGGCTAATC-TAM | 417-440  454-475  540-561 | [8] |

**References**

1. Welinder-Olsson C, Dotevall L, Hogevik H, et al. Comparison of broad-range bacterial PCR and culture of cerebrospinal fluid for diagnosis of community-acquired bacterial meningitis. Clin Microbiol Infect. 2007;13(9):879-86.

2. Niesters HG, van Esser J, Fries E, et al. Development of a real-time quantitative assay for detection of Epstein-Barr virus. J Clin Microbiol. 2000;38(2):712-5.

3. Iacobaeus E, Hopia L, Khademi M, et al. Analysis of JC virus DNA in NPSLE patients treated with different immunomodulatory agents. Lupus. 2013;22(3):307-11.

4. Namvar L, Olofsson S, Bergstrom T, Lindh M. Detection and typing of Herpes Simplex virus (HSV) in mucocutaneous samples by TaqMan PCR targeting a gB segment homologous for HSV types 1 and 2. J Clin Microbiol. 2005;43(5):2058-64.

5. Persson A, Bergstrom T, Lindh M, Namvar L, Studahl M. Varicella-zoster virus CNS disease--viral load, clinical manifestations and sequels. J Clin Virol. 2009;46(3):249-53.

6. Mariani BD, Martin DS, Levine MJ, Booth RE, Jr., Tuan RS. The Coventry Award. Polymerase chain reaction detection of bacterial infection in total knee arthroplasty. Clin Orthop Relat Res. 1996(331):11-22.

7. Fredricks DN, Relman DA. Improved amplification of microbial DNA from blood cultures by removal of the PCR inhibitor sodium polyanetholesulfonate. J Clin Microbiol. 1998;36(10):2810-6.

8. Andersson ME, Olofsson S, Lindh M. Comparison of the FilmArray assay and in-house real-time PCR for detection of respiratory infection. Scand J Infect Dis. 2014;46(12):897-901.
