# Supplementary material for: Optimization of cerebrospinal fluid microbial DNA metagenomic sequencing diagnostics
Source: Sci Rep. 2022 Mar 1;12:3378. doi: 10.1038/s41598-022-07260-x (PMC8888594; doi:10.1038/s41598-022-07260-x)
Supplement: Supplementary file 3 — Supplementary Information 3. [file 41598_2022_7260_MOESM3_ESM.docx]

**Additional Table 1.** Clinical data.

| Sample | Age/Sex | Diagnosis | Verified pathogen | Confirmatory testing | CSF cell count (×10^6^/l) | | | | | Clinical Data | |
| --- | --- | --- | --- | --- | --- | --- | --- | --- | --- | --- | --- |
|  |  |  |  | (Geq/ml) | Ery | Lym | Mon | NeNeu | Pol | |  |
| 1 | 60/F | Encephalitis | HSV1 | qPCR (1.0x10^4^) | 1 |  | 40 |  | 1 | | Fever, dizziness, fainting, confusion, convulsions, pathological Romberg’s sign |
| 2 | 32/M | Meningitis | VZV | qPCR (3.9x10^5^) | 2 | 254 | 18 | 0 |  | | Fever, dizziness, headache, photosensitivity, nausea, vomiting, common cold symptoms, blisters dorsal and ventral left flank, diplopia left eye, pathological Romberg’s sign, neck stiffness. |
| 3 | 24/M | Meningitis | VZV | qPCR (1.9x10^5^) | 92 | 17 | 0 | 0 |  | | Secretory otitis media ongoing healing, common cold symptoms, headache, vomiting |
| 4 | N/A | N/A | JC polyomavirus | qPCR (1.9x10^5^) | N/A | | | | | | N/A |
| 5 | N/A | N/A | JC Polyomavirus | qPCR (4.3x10^3^) | N/A | | | | | | N/A |
| 6 | 65/F | Meningitis  Acute Mastoiditis, Pneumoniae | *S. pneumoniae* | Cultivation and 16S rRNA gene Sanger Seq | 5405 |  | 10 |  | 45 | | Fever, unconsciousness, sore ears, petechiae-lower extremities, positive Babinski’s sign, neck stiffness |
| 7 | 55/M | Meningitis, Otitis media | *S. pneumoniae*  EBV | 16S rRNA gene Sanger seq qPCR (3.7x10^2^) | 112 | 76 | 265 | 723 |  | | Fever, dizziness, headache, common cold symptoms, obtundation, confusion, perforated otitis media, sunset glance, enlarged pupil, weakness on left side, neck stiffness |
| 8 | 37/M | Viral meningitis | Enterovirus | qPCR (6.6x10^4^) | <5 | 65 | 26 | 4 |  | | Headache, photosensitivity, common cold symptoms, fainting, pathological Romberg's sign, pathological finger-nose test |
| 9 | 11/M | Meningoencephalitis | Enterovirus EBV | qPCR (5.8x10^4^) qPCR (4.1x10^2^) | 13 |  | 484 |  | 330 | | Fever, dizziness, headache, photosensitivity, nausea, memory loss, difficulty feeling touch – lower leg |
| 10 | 83/F | Encephalitis | EBV VZV | qPCR (1.9x10^3^) qPCR (4.7x10^3^) | 106 | 160 | 19 | 2 |  | | Fever, constitutional symptoms, varicella zoster, right hemiparesis, neck stiffness |
| 11 | 86/F | Shingles (Orthostatism) | EBV VZV | qPCR (5.0 x10^1^) qPCR (2.9x10^3^) | 27 | 67 | 23 | <3 |  | | Dizziness, nausea, initial fluctuating confusion, varicella zoster right leg, pathological finger-nose test |
| 12 | 57/F | Meningitis | EBV Yeast sp. | qPCR (9.1 x10^2^) Cultivation and Filmarray | 13340 |  | 161 |  | 3 | | Dizziness, headache, blurred vision, falling tendency, dragging walk, weakness left leg, pathological Romberg’s sign |
| 13 | 52/F | CNS Hodgkin's lymphoma | EBV | qPCR (1.9x10^3^) | 5 | 26 | <3 | <3 |  | | Dizziness, nausea, vomiting, constitutional symptoms, somnolence, left hemiparesis, dysarthria |
| 14 | 5/M | Neuroborreliosis | EBV | qPCR (3.7x10^2^) | 403 | 178 | 49 | 26 |  | | Fever, headache, nausea, vomiting, sore neck, previous foul bite without rash |
| 15 | 67/M | Viral encephalitis | EBV | qPCR (3.2x10^2^) | <5 | 32 | 12 | <3 |  | | Confusion, dysphasia with paraphasia and neologisms, pathological finger-nose test |
| 16 | 14/M | Encephalitis, Staphylococcal sepsis, Viral pneumoniae, T-ALL | EBV | qPCR (2.7x10^2^) | <5 | 4 | <3 | <3 |  | | Fever, constitutional symptoms, confusion, slurred speech |
| 17 | 30/M | Postoperative neurosurgical infection | EBV | qPCR (1.6x10^2^) | 220 |  | 300 |  | 148 | | Fever, dizziness, headache, nausea |
| 18 | 74/M | No infection | EBV | qPCR (1.6x10^2^) | 3 | <3 | <3 | 0 |  | | Headache, nausea, vomiting, constitutional symptoms, neck stiffness |
| 19 | 21/F | Encephalitis | EBV | qPCR (8.1 x10^1^) | <5 | 31 | <3 | <3 |  | | Fever, dizziness, headache, common cold symptoms, nausea, vomiting, confusion, convulsions (mycoplasma serum IgM positive and nasopharyngeal swab PCR positive) |
| 20 | 88/F | Encephalitis | EBV | qPCR (5.1 x10^1^) | <5 | 14 | <3 | <3 |  | | Fever, dizziness, nausea, vomiting, constitutional symptoms, depersonalization, wide-range walk, tendency falling backwards, dysarthria, neck stiffness |
| 21 | 72/F | Neuromyelitis optica | EBV | qPCR (5.0 x10^1^) | 91 |  | 8 |  | 1 | | Dermatitis, leg paresis, dissociated feeling, impairment, weakness |

*Geq: Genome equivalents, Ery: erythrocytes, Lym: lymphocytes, Mon: monocytes, Neu: neutrophils, Pol: polycytes. HSV1: Herpes simplex virus 1, VZV: Varicella Zoster virus, EBV: Epstein-Barr virus, T-ALL: T-cell acute lymphoblastic leukemia*

**Additional Table 2.** Datasets species classification

| Sample | |  | | PaRCA | | | | | | | | | | | | | Kraken2 | | | | | | | | | | | | | | | Centrifuge* | | | | | | | | | | | | | CosmosID^#^ | | | | | | | | | | | | | | | | | |  |
| --- | --- | --- | --- | --- | --- | --- | --- | --- | --- | --- | --- | --- | --- | --- | --- | --- | --- | --- | --- | --- | --- | --- | --- | --- | --- | --- | --- | --- | --- | --- | --- | --- | --- | --- | --- | --- | --- | --- | --- | --- | --- | --- | --- | --- | --- | --- | --- | --- | --- | --- | --- | --- | --- | --- | --- | --- | --- | --- | --- | --- | --- | --- | --- |
|  |  | Total reads | Classified Reads | | | | Human Reads | Virus Reads | | Bacteria Reads | | Viral and bacterial species (amount) | | % species  > 0.01% limit† | | % species >0.01% limit after control† | | | Classified Reads | | Human Reads | Virus Reads | | Bacteria Reads | | Viral and bacterial species (amount) | | % species  > 0.01% limit† | | % species >0.01% limit after control† | | | | Classified Reads | Human Reads | | Virus Reads | | Bacteria Reads | | Viral and bacterial species (amount) | | % species  > 0.01% limit† | | | % species >0.01% limit after control† | | Classified Reads | | Human Reads | | Virus Reads | | Bacteria Reads | | Viral and bacterial species (amount) | | % species  > 0.01% limit† | | | % species >0.01% limit after control† | | |
| 1 | 16,155,106 | | | | **15,240,521** | 15,101,081 | | | 4,372 | | 195,129 | | 281 | | 8.5 | 3.6 | | **15,859,247** | | 15,706,386 | | | 117 | | 150,227 | | 515 | | 2.5 | | 0.8 | | **17,250,776** | | | 17,064,379 | | 149 | | 174,726 | | 1598 | | 2.8 | | | 1.5 | | **16,155,106** | | N/A | | 226 | | 59,618 | | 11 | | 54.2 | 18.2 | |  |  |
| 2 | 14,278,116 | | | | **13,280,177** | 13,232,349 | | | 4,431 | | 24,125 | | 65 | | 13.8 | 3.1 | | **13,820,286** | | 13,817,757 | | | 259 | | 605 | | 380 | | 2.9 | | 2.6 | | **14,862,675** | | | 14,850,476 | | 269 | | 1,262 | | 851 | | 2.0 | | | 1.9 | | **14,275,166** | | N/A | | 356 | | 1,413 | | 3 | | 100 | 100 | |  |  |
| 3 | 16,041,251 | | | | **14,924,255** | 14,859,234 | | | 6,755 | | 36,199 | | 98 | | 9.2 | 2.0 | | **15,522,840** | | 15,512,386 | | | 2,273 | | 1,526 | | 411 | | 1.9 | | 1.0 | | **16,699,082** | | | 16,673,106 | | 2,321 | | 2,131 | | 1003 | | 3.8 | | | 3.0 | | **16,041,251** | | N/A | | 2,393 | | 1,910 | | 4 | | 100 | 100 | |  |  |
| 4 | 12,703,224 | | | | **11,338,709** | 6,797,953 | | | 29,530 | | 4,475,933 | | 743 | | 21.7 | 2.3 | | **11,728,272** | | 7,143,611 | | | 24,242 | | 4,577,006 | | 1299 | | 8.5 | | 0.8 | | **13,348,386** | | | 7,928,803 | | 25,421 | | 5,284,937 | | 2580 | | 8.0 | | | 3.1 | | **12,703,224** | | N/A | | 25,986 | | 1,754,615 | | 16 | | 68.8 | 31.3 | |  |  |
| 5 | 12,165,130 | | | | **10,525,017** | 4,231,556 | | | 5,576 | | 6,265,762 | | 720 | | 21.5 | 0.8 | | **10,923,115** | | 4,498,521 | | | 878 | | 6,422,262 | | 1413 | | 8.6 | | 0.4 | | **12,451,198** | | | 4,948,147 | | 1,656 | | 7,294,357 | | 2845 | | 7.3 | | | 2.6 | | **12,165,130** | | N/A | | 2,887 | | 2,549,867 | | 9 | | 77.8 | 66.7 | |  |  |
| 6 | 14,957,135 | | | | **12,650,219** | 11,396,742 | | | 6,437 | | 1,153,776 | | 369 | | 11.7 | 5.7 | | **14,486,711** | | 13,517,631 | | | 11 | | 963,584 | | 928 | | 2.0 | | 0.6 | | **18,748,448** | | | 17,194,162 | | 100 | | 1,526,405 | | 2084 | | 4.8 | | | 4.1 | | **14,957,135** | | N/A | | 1,275 | | 104,057 | | 14 | | 57.1 | 28-6 | |  |  |
| 7 | 17,365,318 | | | | **16,152,332** | 16,069,006 | | | 6,081 | | 50,834 | | 112 | | 9.8 | 0.9 | | **16,923,847** | | 16,904,670 | | | 7 | | 16,840 | | 460 | | 1.7 | | 1.1 | | **18,419,777** | | | 18,389,723 | | 22 | | 25,307 | | 1039 | | 2.1 | | | 1.4 | | **17,365,318** | | N/A | | 171 | | 4,012 | | 5 | | 20 | 20 | |  |  |
| 8 | 13,417,152 | | | | **12,532,220** | 12,383,644 | | | 3,657 | | 121,529 | | 244 | | 7.8 | 3.7 | | **13,134,090** | | 13,046,745 | | | 8 | | 84,987 | | 504 | | 2.6 | | 0.2 | | **14,371,346** | | | 14,261,140 | | 35 | | 98,431 | | 1546 | | 2.1 | | | 1.5 | | **13,417,152** | | N/A | | 149 | | 33,851 | | 7 | | 42.9 | 0 | |  |  |
| 9 | 11,006,034 | | | | **10,128,505** | 10,086,217 | | | 2,791 | | 25,089 | | 95 | | 8.4 | 0 | | **10,634,909** | | 10,634,909 | | | 9 | | 4,147 | | 404 | | 2.0 | | 1.2 | | **11,408,918** | | | 11,400,094 | | 20 | | 5,319 | | 962 | | 2.0 | | | 1.1 | | **11,006,034** | | N/A | | 94 | | 2,555 | | 4 | | 0 | 0 | |  |  |
| 10 | 9,600,578 | | | | **8,934,196** | 8,859,290 | | | 2,840 | | 59,202 | | 208 | | 7.7 | 1.4 | | **9,322,094** | | 9,277,062 | | | 24 | | 242,141 | | 553 | | 2.9 | | 1.4 | | **10,019,619** | | | 9,962,846 | | 43 | | 50,731 | | 1537 | | 2.3 | | | 1.2 | | **9,600,578** | | N/A | | 113 | | 17,385 | | 9 | | 66.7 | 33.3 | |  |  |
| 11 | 9,569,552 | | | | **8,900,918** | 8,792,316 | | | 2,620 | | 93,511 | | 227 | | 7.5 | 3.1 | | **9,283,282** | | 9,204,476 | | | 20 | | 77,768 | | 514 | | 2.5 | | 1.6 | | **9,984,472** | | | 9,885,888 | | 51 | | 89,799 | | 1539 | | 3.0 | | | 1.4 | | **9,535,729** | | N/A | | 123 | | 30,691 | | 7 | | 71.4 | 14.3 | |  |  |
| 12 | 12,244,759 | | | | **11,413,586** | 10,932,187 | | | 4,997 | | 409,339 | | 356 | | 12.4 | 4.2 | | **11,919,736** | | 11,566,102 | | | 26 | | 342,404 | | 769 | | 4.3 | | 0.9 | | **13,796,034** | | | 13,377,407 | | 82 | | 379,079 | | 1861 | | 3.6 | | | 1.5 | | **12,244,759** | | N/A | | 202 | | 133,832 | | 8 | | 62.5 | 0 | |  |  |
| 13 | 11,791,941 | | | | **10,831,390** | 10,701,867 | | | 3,548 | | 110,867 | | 161 | | 11.2 | 5.6 | | **11,387,096** | | 11,291,000 | | | 98 | | 94,739 | | 732 | | 2.2 | | 1.5 | | **12,260,830** | | | 12,143,605 | | 115 | | 107,741 | | 1852 | | 2.1 | | | 1.1 | | **11,791,941** | | N/A | | 239 | | 36,766 | | 12 | | 58.3 | 25 | |  |  |
| 14 | 15,668,502 | | | | **14,591,084** | 14,460,476 | | | 4,308 | | 107,011 | | 243 | | 6.2 | 0.4 | | **15,213,606** | | 15,128,599 | | | 10 | | 83,995 | | 655 | | 1.8 | | 1.2 | | **16,264,830** | | | 16,259,511 | | 35 | | 97,425 | | 1807 | | 2.0 | | | 1.2 | | **15,668,502** | | N/A | | 149 | | 34,593 | | 7 | | 42.9 | 0 | |  |  |
| 15 | 13,852,556 | | | | **12,977,595** | 12,790,541 | | | 3,836 | | 164,700 | | 272 | | 7.4 | 3.7 | | **13,499,122** | | 13,356,211 | | | 19 | | 141,659 | | 645 | | 2.2 | | 1.4 | | **14,487,022** | | | 14,303,887 | | 49 | | 163,618 | | 1843 | | 2.6 | | | 1.0 | | **13,809,049** | | N/A | | 172 | | 56,224 | | 10 | | 50 | 20 | |  |  |
| 16 | 11,061,515 | | | | **10,165,144** | 9,347,773 | | | 3,159 | | 798,825 | | 460 | | 12.2 | 8.0 | | **10,678,731** | | 9,880,623 | | | 241 | | 769,897 | | 898 | | 7.1 | | 5.8 | | **11,604,819** | | | 10,670,040 | | 351 | | 918,003 | | 2337 | | 5.0 | | | 1.1 | | **11,061,515** | | N/A | | 511 | | 315,290 | | 11 | | 72.7 | 54.5 | |  |  |
| 17 | 9,993,372 | | | | **9,264,766** | 9,217,416 | | | 2,976 | | 30,329 | | 168 | | 6.5 | 0.6 | | **9,690,977** | | 9,673,204 | | | 15 | | 16,641 | | 422 | | 2.1 | | 1.2 | | **10,417,701** | | | 10,294,763 | | 27 | | 19,439 | | 1054 | | 1.7 | | | 1.8 | | **9,993,372** | | N/A | | 101 | | 7,717 | | 5 | | 80 | 20 | |  |  |
| 18 | 19,727,930 | | | | **18,336,989** | 17,070,118 | | | 4,779 | | 1,234,359 | | 536 | | 9.9 | 6.3 | | **19,159,304** | | 17,942,646 | | | 27 | | 1,215,263 | | 1115 | | 4.8 | | 4.2 | | **20,761,857** | | | 19,342,338 | | 127 | | 1,391,846 | | 2549 | | 4.0 | | | 4.7 | | **19,727,930** | | N/A | | 480 | | 499,185 | | 8 | | 75 | 75 | |  |  |
| 19 | 9,611,223 | | | | **8,942,217** | 8,867,132 | | | 2,858 | | 60,693 | | 200 | | 6.5 | 0.5 | | **9,347,387** | | 9,296,863 | | | 5 | | 49,591 | | 476 | | 2.3 | | 0.8 | | **10,034,970** | | | 9,972,248 | | 22 | | 57,726 | | 1309 | | 2.1 | | | 1.0 | | **9,611,223** | | N/A | | 111 | | 19,746 | | 6 | | 83.3 | 83.3 | |  |  |
| 20 | 11,375,823 | | | | **10,526,555** | 10,242,680 | | | 2,955 | | 264,731 | | 314 | | 9.6 | 5.1 | | **11,061,201** | | 10,818,124 | | | 10 | | 242,141 | | 736 | | 2.6 | | 1.9 | | **11,927,331** | | | 11,640,347 | | 24 | | 270,426 | | 1956 | | 2.5 | | | 3.7 | | **11,375,823** | | N/A | | 147 | | 95,885 | | 8 | | 87.5 | 75 | |  |  |
| 21 | 10,687,870 | | | | **10,016,450** | 9,709,562 | | | 3,836 | | 289,961 | | 338 | | 9.8 | 6.8 | | **10,398,272** | | 10,108,840 | | | 17 | | 288,096 | | 666 | | 3.9 | | 3.6 | | **11,207,425** | | | 10,869,174 | | 68 | | 327,877 | | 2002 | | 3.7 | | | 1.0 | | **10,687,870** | | N/A | | 211 | | 119,483 | | 10 | | 70 | 60 | |  |  |

** The total of both leaf and genus levels were merged from the Centrifuge reports, leading to higher amounts of total classified reads. ^#^Human reads were not provided by CosmosID, hits instead of read.* † >0.01% limit used for bacterial species, removal of species with control includes both bacterial and viral species.

**Additional Table 3.** Pathogen detection by bioinformatic classifier.

| Sample |  |  |  | PaRCA | | Kraken2 | | Centrifuge* | | CosmosID | | BLAST |
| --- | --- | --- | --- | --- | --- | --- | --- | --- | --- | --- | --- | --- |
|  | Verified Pathogen | Method (Geq/ml) | Total reads Ion Torrent | Reads/total class. reads | ppm | Reads/total class. reads | ppm | Reads/total class. reads | ppm | Reads/total  class. reads | ppm | Unique Reads |
| 1 | HSV1 | qPCR (1.0x10^4^) | 16,155,106 | 97/ 15,240,521 | 6.4 | 105/ 15,859,247 | 6.6 | 107/ 17,250,776 | 6.2 | 107/ 16,155,106 | 6.6 | 108 |
| 2 | VZV | qPCR (3.9x10^5^) | 14,278,116 | 213/ 13,280,177 | 16.0 | 219/ 13,820,286 | 15.9 | 223/ 14,862,675 | 15.0 | 211/ 14,275,166 | 14.9 | 213 |
| 3 | VZV | qPCR (1.9x10^5^) | 16,041,251 | 2,196/ 14,924,255 | 147.1 | 2,234/ 15,522,840 | 143.9 | 2,251/ 16,699,082 | 134.8 | 2,170/ 16,041,251 | 135.3 | 2197 |
| 4 | JCV | qPCR (1.9x10^5^) | 12,703,224 | 23,766/ 11,338,709 | 2,096.0 | 24,018/ 11,728,272 | 2,047.9 | 24,190/ 13,348,386 | 1,812.2 | 22,318/12,703,224 | 1,756.9 | 2, 847 |
| 5 | JCV | qPCR (4.3x10^3^) | 12,165,130 | 496/ 10,525,017 | 47.1 | 512/ 10,923,115 | 46.9 | 515/ 12,451,198 | 41.4 | 484/ 12,165,130 | 39.8 | 498 |
| 6 | *SP* | Cultivation & 16S rRNA  gene Seq | 14,957,135 | 766,744/ 12,650,219 | 60,611.1 | 699,662/ 14,486,711 | 48,296.8 | 575,646/ 18,748,448 | 30,703.7 | 701,304/ 14,957,135 | 46,887.6 | 643,083 |
| 7 | *SP E*BV | 16S rRNA gene Seq qPCR (3.7x10^2^) | 17,365 318 | 12,988/ 16,152,332 0/ 16,152,332 | 804.1 - | 11,762/ 16,923,847 0/ 16,923,847 | 695.0 - | 12,511/ 18,419,777 0/ 18,419,777 | 679.2 - | 12,277/ 17,365,318 0/ 17,365,318 | 707.0 - | 12,274 0 |
| 8 | EV | qPCR (6.6x10^4^) | 13,417,152 | 0/ 12,532,220 | - | 0/ 13,134,090 | - | 0/ 14,371,346 | - | 0/ 13,417,152 | - | 0 |
| 9 | EV EBV | qPCR (5.8x10^4^) qPCR (4.1x10^2^) | 11,006,034 | 0/ 10,128,505 0/ 10,128,505 | - - | 0/ 10,634,909 0/ 10,634,909 | - - | 0/ 11,408,918 0/ 11,408,918 | - - | 0/ 11,006,034 0/ 11,006,034 | - - | 0 0 |
| 10 | EBV VZV | qPCR (1.9x10^3^) qPCR (4.7x10^3^) | 9,600,578 | 10/ 8,934,196 7/ 8,934,196 | 1.1 0.8 | 9/ 9,322,094 7/ 9,322,094 | 1.0 0.8 | 9/ 10,019,619 7/ 10,019,619 | 0.9 0.7 | 8/ 9,600,578 7/ 9,600,578 | 0.8 0.7 | 9 7 |
| 11 | EBV VZV | qPCR (5.0 x10^1^) qPCR (2.9x10^3^) | 9,569,552 | 0/ 8,900,918 15/ 8,900,918 | - 1.7 | 0/ 9,283,282 15/ 9,283,282 | - 1.6 | 0/ , 984,472 15/ 9,984,472 | - 1.5 | 0/ 9,535,729 12/ 9,535,729 | - 1.2 | 0 15 |
| 12 | EBV Yeast | qPCR (9.1 x10^2^) Cultivation & Filmarray | 12,244,759 | 0/11,413,586 0/11,413,586 | - - | 0/ 11,919,736 0/ 11,919,736 | - - | 0/ 13,796,034 0/ 13,796,034 | - - | 0/ 12,244,759 0/ 12,244,759 | - - | 0 0 |
| 13 | EBV | qPCR (1.9x10^3^) | 11,791,941 | 81/ 10,831,390 | 7.5 | 85/ 11,387,096 | 7.5 | 82/ 12,260,830 | 6.7 | 79/ 11,791,941 | 6.7 | 82 |
| 14 | EBV | qPCR (3.7x10^2^) | 15,668,502 | 0/ 14,591,084 | - | 0/ 15,213,606 | - | 0/ 16,264,830 | - | 0/ 15,668,502 | - | 0 |
| 15 | EBV | qPCR (3.2x10^2^) | 13,852,556 | 6/ 12,977,595 | 0.5 | 6/ 13,499,122 | 0.4 | 6/ 14,487,022 | 0.4 | 6/ 13,809,049 | 0.5 | 6 |
| 16 | EBV | qPCR (2.7x10^2^) | 11,061,515 | 232/ 10,165,144 | 22.8 | 228/ 10,678,731 | 21.4 | 225/ 11,604,819 | 19.4 | 213/ 11,061,515 | 21.2 | 223 |
| 17 | EBV | qPCR (1.6x10^2^) | 9,993,372 | 11/ 9,264,766 | 1.2 | 10/ 9,690,977 | 1.0 | 11/ 10,417,701 | 1.1 | 11/ 9,993,372 | 1.1 | 11 |
| 18 | EBV | qPCR (1.6x10^2^) | 19,727,930 | 0/ 18,336,989 | - | 0/19,159,304 | - | 0/ 20,761,857 | - | 0/ 19,727,930 | - | 0 |
| 19 | EBV | qPCR (8.1 x10^1^) | 9,611,223 | 0/ 8,942,217 | - | 0/ 9,347,387 | - | 0/ 10,034,970 | - | 0/ 9,611,223 | - | 0 |
| 20 | EBV | qPCR (5.1 x10^1^) | 11,375,823 | 0/ 10,526,555 | - | 1/ 11,061,201 | 0.1 | 1/ 11,927,331 | 0.1 | 0/ 11,375,823 | - | 1 |
| 21 | EBV | qPCR (5.0 x10^1^) | 10,687,870 | 8/ 10,016,450 | 0.8 | 8/ 10,398,272 | 0.8 | 8/ 11,207,425 | 0.7 | 8/ 10,687,870 | 0.8 | 9 |

*Reads/total class. reads: Pathogen reads/total reads classified by bioinformatical pipeline. ppm: parts per million, abundance of pathogen per millions of total classified reads. JCV: JC polyomavirus,
SP: Streptococcus pneumonia, EV: Enterovirus *The total of both leaf and genus levels were incorporated in the Centrifuge reports, leading to higher amounts of total classified reads, however this
did not affect the final amount of pathogen reads.*

**Additional Table 6.** Cell control reproducibility.

|  |  | **PaRCA** | | | **Kraken2** | | | **Centrifuge** | | | **CosmosID** | | |
| --- | --- | --- | --- | --- | --- | --- | --- | --- | --- | --- | --- | --- | --- |
| Control | ID# | Classified reads | EBV Reads | ppm (mean±SD) | Classified reads | EBV Reads | ppm (mean±SD) | Classified reads | EBV Reads | ppm (mean±SD) | Classified reads | EBV Reads | ppm (mean±SD) |
| **Namalwa**  **Cell line** | **#1** | 14,441,248 | 16,584 | **1006±225** | 15,085,293 | 16,894 | **955±199** | 16,136,669 | 16,952 | **920±210** | 15,561,865 | 16,040 | **913±201** |
|  | **#2** | 11,757,402 | 14,115 |  | 12,208,229 | 13,227 |  | 13,008,822 | 14,341 |  | 12,505,333 | 13,672 |  |
|  | **#3** | 14,385,091 | 10,095 |  | 15,021,622 | 10,232 |  | 16,081,341 | 10,212 |  | 15,325,051 | 9,828 |  |
|  | **#4** | 7,490,706 | 7,280 |  | 7,788,053 | 7,289 |  | 8,291,564 | 7,387 |  | 7,970,628 | 7,063 |  |
| **P3HR1**  **Cell line** | **#1** | 12,641,680 | 14,798 | **1335±207** | 13,260,465 | 15,133 | **1210±183** | 14,189,956 | 15,155 | **1215±192** | 13,693,487 | 14,265 | **1191±176** |
|  | **#2** | 9,483,576 | 12,753 |  | 9,847,965 | 12,924 |  | 10,520,818 | 12,930 |  | 10,060,502 | 12,307 |  |
|  | **#3** | 11,421,831 | 12,410 |  | 12,029,195 | 12,665 |  | 12,962,700 | 12,677 |  | 12,388,273 | 12,010 |  |
|  | **#4** | 9,191,839 | 13,973 |  | 9,634,063 | 14,263 |  | 10,348,117 | 14,271 |  | 9,938,198 | 12,523 |  |
|  | **#5** | 12,761,346 | 19,829 |  | 13,243,991 | 14,099 |  | 14,129,912 | 20,066 |  | 13,591,011 | 19,157 |  |
| **Water** |  | 3,789,807 | 0 | **N/A** | 3,864,601 | 0 | **N/A** | 4,517,577 | 0 | **N/A** | 4,583,998 | 0 | **N/A** |

*ID#: cell controls extracted, and sequenced at different time-points. EBV: Epstein-Barr virus, N/A Not applicable, ppm: reads per million of total classified reads*
