# Supplementary material for: Optimization of cerebrospinal fluid microbial DNA metagenomic sequencing diagnostics
Source: Sci Rep. 2022 Mar 1;12:3378. doi: 10.1038/s41598-022-07260-x (PMC8888594; doi:10.1038/s41598-022-07260-x)
Supplement: Supplementary file 5 — Supplementary Information 5. [file 41598_2022_7260_MOESM5_ESM.docx]

**Overview additional tables and figure legends**

**Additional table 1.** Clinical data (docx)

**Additional table 2.** Dataset species classification (docx)

**Additional table 3.** Pathogen detection by bioinformatic classifier (docx)

**Additional table 4.** Patient report with estimation of sensitivity for pathogens (xlsx)
**Additional table 5.** Species identified in bioinformatic classifiers (xlsx)

**Additional table 6.** Cell control reproducibility (docx)

**Additional method.** Description of clinical methodology used for comparison (docx)

**Additional figure 1.** Enterovirus samples (pdf)

Results of viral species detected in RNA sequencing datasets of sample 8 and sample 9 in PaRCA. Reads mapping to enterovirus was found amongst virus lists in both specimens.

**Additional figure 2. Detected pathogens in bioinformatic classifiers**

Number of viral (a) and bacterial species (b) classified in each of the samples and controls using the different bioinformatic classifiers. Dark blue bars show the total number of species classified, bright blue bars show the number of bacterial species over the fraction cutoff (0.01% of the dataset), light blue bars show number of species not removed using controls.

**Additional figure 3.** Coverage density plot of microbial species in CSF samples  (pdf)

Reads from samples not shown in main figure mapped to reference genomes of (a) VZV (NC_001348), (b) JCV (NC_00196), (c) *S. pneumoniae* (NC_003098), (d, f) VZV (NC_001348), and (e, g-j) EBV (NC_007605) using CLC Genomics Workbench. Number of reads (y-axis) at each nucleotide position of the genome (x-axis) depicted in blue. Dark blue represents peak, bright blue average and light blue minimum coverage for respective sections of the genome.

**Additional figure 4.** Cell control coverage density plot and reproducibility (pdf)

Coverage analysis of EBV reads detected in cell controls Namalwa (a) and P3HR1 (b) mapped to EBV reference genome (NC_007605) using CLC Genomics Workbench. Number of reads (y-axis) at each nucleotide position of the genome (x-axis) depicted in blue. Dark blue represents peak, bright blue average and light blue minimum coverage for respective sections of the genome. EBV read fraction of sequencing library shown as parts per million reads (ppm) in each of the cell line controls for each of the bioinformatic classifiers (c), n=4 (Namalwa) or n=5 (P3HR1). Kruskal-Wallis test with Dunn's multiple comparisons show no significant difference between the pipelines.

**Additional figure 5.** Coverage analysis for unexpected findings (pdf)

Reads from samples with ambiguous findings mapped to reference genomes of EBV NC_007605 (a-b), Human Mastadenovirus C (MAVC) NC_001405 (c), Human Papillomavirus 98 (HPV98) FM_955837.2 (d), Anellovirus MH_649255.1 (e), and HCV NC_004102.1 (f), using CLC Genomics Workbench.
